# The Relationship Between Electronic Health Literacy and Health-Related Quality of Life Among Chinese Older Adults: Cross-Sectional Study

**DOI:** 10.2196/84700

**Published:** 2026-03-24

**Authors:** Yongqiang Wang, Baozhen Dai, Jiazhen Yao

**Affiliations:** 1 Health Policy and Healthcare Security Research Center, Southeast University, No. 87 Dingjiaqiao, Nanjing, Jiangsu, 210009, China, 86 18761863696; 2 Ageing-Responsive Civilization Think Tank, Nanjing, Jiangsu, China

**Keywords:** electronic health literacy, health-related quality of life, older adult, attitudes toward own aging, self-efficacy

## Abstract

**Background:**

The rapid digitalization of health care has reshaped access to medical services. However, older adults often remain disadvantaged due to the digital divide. Electronic health literacy (EHL) is increasingly recognized as a determinant of health-related quality of life (HRQoL); however, its mechanisms and subgroup differences in China remain underexplored.

**Objective:**

This study aimed to examine the association between EHL and multidimensional HRQoL among Chinese older adults, with a focus on the mediating roles of attitudes toward own aging (ATOA) and self-efficacy (SE), and heterogeneity by age, residence, and lifestyle.

**Methods:**

A cross-sectional survey (July-November 2024) included 8364 adults aged ≥55 years from 4 provinces using stratified multistage sampling. HRQoL was measured by physical health (PH), mental health (MH), and life satisfaction (LS). EHL was assessed with the eHealth Literacy Scale (eHEALS), ATOA with the Philadelphia Geriatric Center Morale Scale subscale, and SE with the General Self-Efficacy Scale. Analyses used seemingly unrelated regressions, PROCESS (Andrew F. Hayes) macro mediation with 5000 bootstraps, and subgroup regressions.

**Results:**

EHL was positively associated with PH (*β*=0.273; *P*<.001), MH (*β*=0.190; *P*<.001), and LS (*β*=0.082; *P*<.001). ATOA and SE significantly mediated these associations (all *P*<.001). For PH, the association was partially mediated by ATOA and SE. The total effect was 0.273 (*P*<.001), with indirect effects accounting for 52.7% of the total. For MH, inconsistent mediation was observed. The direct effect was negative (*β*=–0.069; *P*<.01), but indirect effects were positive, yielding a positive total effect. For LS, the effect was fully mediated by ATOA and SE. Subgroup analyses showed stronger effects in the younger-old adults (PH: *β*=0.288, MH: *β*=0.188, LS: *β*=0.089; all *P*<.001), urban residents (PH: *β*=0.237, MH: *β*=0.214, LS: *β*=0.083; all *P*<.001), and nonexercisers (PH: *β*=0.322; *P*<.001).

**Conclusions:**

EHL is strongly associated with HRQoL among Chinese older adults. Its effects on MH and LS operate primarily through psychosocial pathways, while PH benefits directly. The findings highlight EHL’s compensatory role, particularly for the younger-old group, rural residents, and nonexercisers, underscoring its importance in digital inclusion and healthy aging policies.

## Introduction

### Background

The accelerating pace of global population aging has made the pursuit of healthy aging a pressing challenge for both public health and social development [1]. Beard et al [2] argue that healthy aging is not merely about extending life expectancy but about maintaining functional ability, psychological well-being, and social participation across the entire lifespan, thereby improving the holistic welfare of older adults. Under this overall objective, health-related quality of life (HRQoL) has become a key comprehensive indicator for measuring its achievement. HRQoL refers to an individual’s subjective assessment of their well-being and ability to perform physical, psychological, and social functions [3]. Considering the landscape of healthy aging, China is undergoing the world’s largest and fastest demographic transition, where high prevalence of chronic diseases, functional decline, and social isolation intertwine, severely constraining the progress of healthy aging [4]. Relevant surveys also indicate that the HRQoL among China’s older adults remains suboptimal [5,6]. Therefore, enhancing HRQoL is not only paramount to achieving healthy aging but also an urgent imperative for addressing the challenges of China’s aging society.

Concurrently, the digital transformation of health and medical service systems is reshaping the way care is delivered [7]. Electronic health services—such as online prescription, telemedicine, electronic health record, and health information platform—have become vital channels for older adults to obtain health resources. These services transcend traditional barriers of time and space, improving accessibility and the efficiency of health management [8]. Yet not all older adults benefit equally. Owing to the persistent digital divide, many face barriers in accessing, evaluating, and using health information, which may exacerbate health inequalities [9]. In this context, electronic health literacy (EHL) has increasingly been recognized as a critical determinant of HRQoL among older adults [10].

EHL refers to the ability to locate, understand, evaluate, and apply health information from electronic media, as well as the capacity to use acquired health information to address health issues [11]. Existing evidence has shown consistent associations between EHL and a range of health outcomes [12-14]. Older adults with higher levels of EHL are more likely to seek health information online, and in doing so, report better self-rated health, improved chronic disease management, and healthier behaviors [15]. EHL has also been associated with improved mental health (MH) and subjective well-being [14]. Nevertheless, important gaps remain, as existing research has focused on self-assessed health as a single measurement tool [16,17], failing to adopt a comprehensive perspective encompassing physical health (PH), MH, and subjective well-being. Furthermore, the mediating mechanisms underlying these associations and the heterogeneity among population subgroups have not been sufficiently explored [16,18].

In China, the “Internet Plus Healthcare” initiative has expanded digital health services nationwide [15]. However, the general level of EHL among older adults remains relatively low, with marked regional and subgroup disparities [19]. Although older adults should be the main beneficiaries of digital health innovations, they often remain a disadvantaged group in practice due to the digital divide [20]. Consequently, empirical investigation into the role of EHL in shaping HRQoL in China remains scarce, highlighting the need to investigate its mechanisms and contextual variations.

### Theoretical Hypotheses

Since the World Health Organization declared 2021-2030 the “Decade of Healthy Aging,” enhancing HRQoL has become the key objective for achieving healthy aging [21]. HRQoL encompasses not only the maintenance of physical functioning but also MH, life satisfaction (LS), and cognitive well-being—positive indicators that extend beyond morbidity or disease prevalence [22]. Within the digital era, the impact of internet use on the health of older adults requires positive mediation through human capital. As a critical form of human capital in the digital health field, EHL directly empowers older adults’ health management practices by enhancing their capacity to discern health information and optimizing pathways to health services, thereby advancing healthy aging [23]. Similarly, EHL is recognized as both a prerequisite for inclusive health innovation and a super determinant of health equity [24]. Systematic reviews have also confirmed a significant positive correlation between EHL and older adults’ health management efficacy [25]. Thus, EHL is not only an essential core health competency for older adults in the digital age but also, due to its enabling role in health management practices and its positive predictive power for health outcomes, emerges as a form of health capital closely linked to HRQoL.

The theory of successful aging emphasizes that older adults’ subjective attitudes toward aging play a critical role in maintaining functional capacity [26,27]. EHL, as a resource for information empowerment and cognitive development, can effectively challenge and mitigate negative attitudes toward aging among older adults. This enables them to view aging as a stage where vitality, learning, and growth remain possible [28]. In turn, positive attitudes toward own aging (ATOA) contribute to the enhancement of older adults’ HRQoL across multiple dimensions. Psychologically, positive ATOA can alleviate aging-related stress responses, strengthen psychological resilience, and reduce the risk of depression [29]. Behaviorally, older adults with positive attitudes are more likely to adopt and maintain health-promoting behaviors to proactively preserve their capabilities [30]. Physiologically, existing research has confirmed that positive self-perceptions of aging exert direct positive physiological effects on cardiovascular and immune functions by alleviating chronic stress [31]. Based on these findings, our study identifies ATOA as a mediating variable to explore the underlying mechanisms.

The conceptual model proposed by Paasche-Orlow and Wolf emphasizes that self-efficacy (SE) serves as a pivotal mediating factor linking health literacy to health outcomes [32]. SE refers to an individual’s subjective judgment and intrinsic confidence in their own capabilities and personal worth within specific contexts [33]. In digital health settings, higher EHL empowers older adults to more effectively access, understand, evaluate, and apply digital health information and services. This process directly enhances their perceived capacity to address health issues. According to social cognitive theory, experiences of successfully mastering and applying relevant skills are key sources of SE. Thus, EHL strengthens older adults’ confidence in managing their health by providing experiences of successfully using digital tools for disease prevention, health management, and self-care [34]. SE theory posits that an individual’s belief in their ability to perform specific tasks is a core determinant of health behaviors [35,36]. Enhanced SE promotes a range of adaptive health behaviors and psychological processes. Older adults with high SE are more likely to adopt proactive health behaviors, adhere to treatment plans, better cope with disease-related stress, and maintain greater psychological resilience. These factors collectively contribute to improvements in both physical and MH, ultimately manifesting as enhanced HRQoL [37]. Thus, our study examines key mechanisms within the relationship by identifying SE as a mediating variable.

Furthermore, the relationship between EHL and HRQoL varies across contexts and individual characteristics. According to health inequality theory, health outcomes are shaped not only by individual factors but also by social determinants [38]. Age reflects significant life course characteristics. Older adults often experience cumulative health decline, cognitive impairment, and increased barriers to digital access and use, making their EHL more likely constrained by physiological and cognitive foundations [39]. Younger-old adults typically possess relatively better physical and mental functioning, along with stronger learning abilities. They may more effectively translate EHL into concrete actions for health management and promotion, thereby demonstrating greater health benefits. Regarding residential environments, urban older adults generally benefit from more comprehensive health care systems, denser social support networks, wider internet and smart device coverage, and higher overall educational attainment and digital literacy [40]. These conditions collectively create an enabling environment conducive to accumulating and converting EHL. In contrast, rural older adults generally face multiple constraints, including low accessibility to medical resources, weak digital infrastructure, limited health information channels, and deep-rooted traditional views on aging, exacerbating health inequalities in the digital age [41]. From the perspective of individual adaptive strategies, lifestyle serves as another crucial moderating factor. According to the Selective Compensation Optimization model, individuals cope with resource changes during aging by selecting goals, optimizing resources, and compensating for losses [42]. For older adults with insufficient exercise, EHL may play a crucial compensatory role by promoting other health behaviors to partially offset health risks stemming from physical inactivity [43,44]. Conversely, for those with established exercise habits—whose health behavior systems are relatively well-developed—EHL’s impact may manifest more as an optimization support. It can enhance the scientific rigor of exercise routines or integrate diverse health resources, yielding relatively stable incremental benefits.

Overall, existing research has yet to establish an integrated research framework. Most studies focus on single mechanisms such as health-promoting behaviors [45], insomnia and psychological distress [46], sense of coherence [47], and SE of health practices [48]. Meanwhile, existing studies lack exploration of the role of social structural factors and individual adaptive strategies as grouping variables [45,49]. Therefore, our study developed an integrated research framework through theoretical synthesis and conceptual framework construction. Therefore, our study integrates theories and constructs a framework encompassing digital empowerment, psychological-behavioral transformation, comprehensive health outcomes, and multilevel contextual moderation. This framework aims to systematically elucidate the mechanisms underlying the functioning of EHL and identify the key entry points for the delivery of targeted interventions. Based on this framework, the study proposes the following hypotheses:

- Hypothesis 1: EHL is positively associated with HRQoL.
- Hypothesis 2a: ATOA mediates the relationship between EHL and HRQoL.
- Hypothesis 2b: SE mediates the relationship between EHL and HRQoL.
- Hypothesis 3a: The association between EHL and HRQoL differs by age.
- Hypothesis 3b: The association between EHL and HRQoL differs by place of residence.
- Hypothesis 3c: The association between EHL and HRQoL differs by exercise behavior.

## Methods

### Study Design and Participants

In 2024, our research team independently conducted a special survey titled Digital Inclusion and Healthy Aging System Development. The questionnaire covered demographic information, digital health literacy, and multidimensional indicators of healthy aging. The inclusion criteria were (1) must be at least 55 years of age at the time of the survey, (2) must have resided continuously within the survey area for no less than 6 months, (3) must possess adequate cognitive and communication abilities, and (4) must voluntarily participate in this study and sign an informed consent form before the survey commencement. Exclusion criteria were (1) severe cognitive or psychiatric impairment preventing effective communication, (2) significant PH issues rendering completion of the survey impossible, (3) temporary residents, and (4) individuals who explicitly refused to sign the informed consent form or withdrew from the study midway.

To ensure high data quality and representativeness, a stratified multistage cluster sampling strategy was adopted, combining purposive and random sampling. In the first stage, based on the digital development index, internet penetration rate, and pilot programs of smart health care for older adults published by the National Bureau of Statistics and the Ministry of Industry and Information Technology, along with regional characteristics, population structure, economic development, and resource distribution, 4 representative provinces or autonomous regions were selected, namely, Jiangsu, Shandong, Hubei, and Guangxi. In the second stage, one city from each province was chosen according to the same stratification criteria, namely, Nanjing, Weifang, Enshi, and Nanning. In the third stage, all districts (or counties) were used as the sampling frame, and random sampling identified Gulou District, Xuanwu District, Qixia District, Jiangning District, Qingzhou County, Jianshi County, and Xixiangtang District. In the fourth stage, taking into account factors such as community population size, urban-rural type, and digital environment variation, a total of 57 communities (or villages) were randomly selected from these districts or counties as the final clusters. Eligible older adults within these units were surveyed. The structural characteristics of the final sample demonstrate that the sampling design achieved balance in economic level, urban-rural type, and digital access status.

Data collection was completed through surveyor-led face-to-face structured interviews. Fieldwork was conducted by a team of trained surveyors who had undergone a standardized training program covering survey objectives, questionnaire content, interview techniques, and ethical considerations.

#### Questionnaire Development and Pretesting

The questionnaire was initially developed in Chinese. To ensure content validity and comprehensibility for the target population, a pretest was conducted among a convenience sample of 200 older adults from nonsampled communities. Based on feedback regarding item clarity, response options, and interview duration, revisions were made to the wording and formatting.

#### Translation

As this study aims for potential international dissemination, the questionnaire was professionally translated into English by bilingual public health researchers following a forward-backward translation procedure to ensure conceptual equivalence. The English version was used for reporting purposes only.

#### Quality Control Procedures

Multiple quality control measures were implemented throughout the data collection process. (1) All surveyors were required to pass a mock interview assessment before fieldwork. (2) On-site supervisors randomly observed approximately 10% of the interviews and provided immediate feedback. (3) Surveyors used tablet computers equipped with a survey application to minimize data entry errors. (4) Supervisors reviewed completed questionnaires daily for completeness and consistency. (5) A random subset of participants was selected for telephone callback to verify key information.

#### Response Rate and Data Cleaning

**Figure 1.**
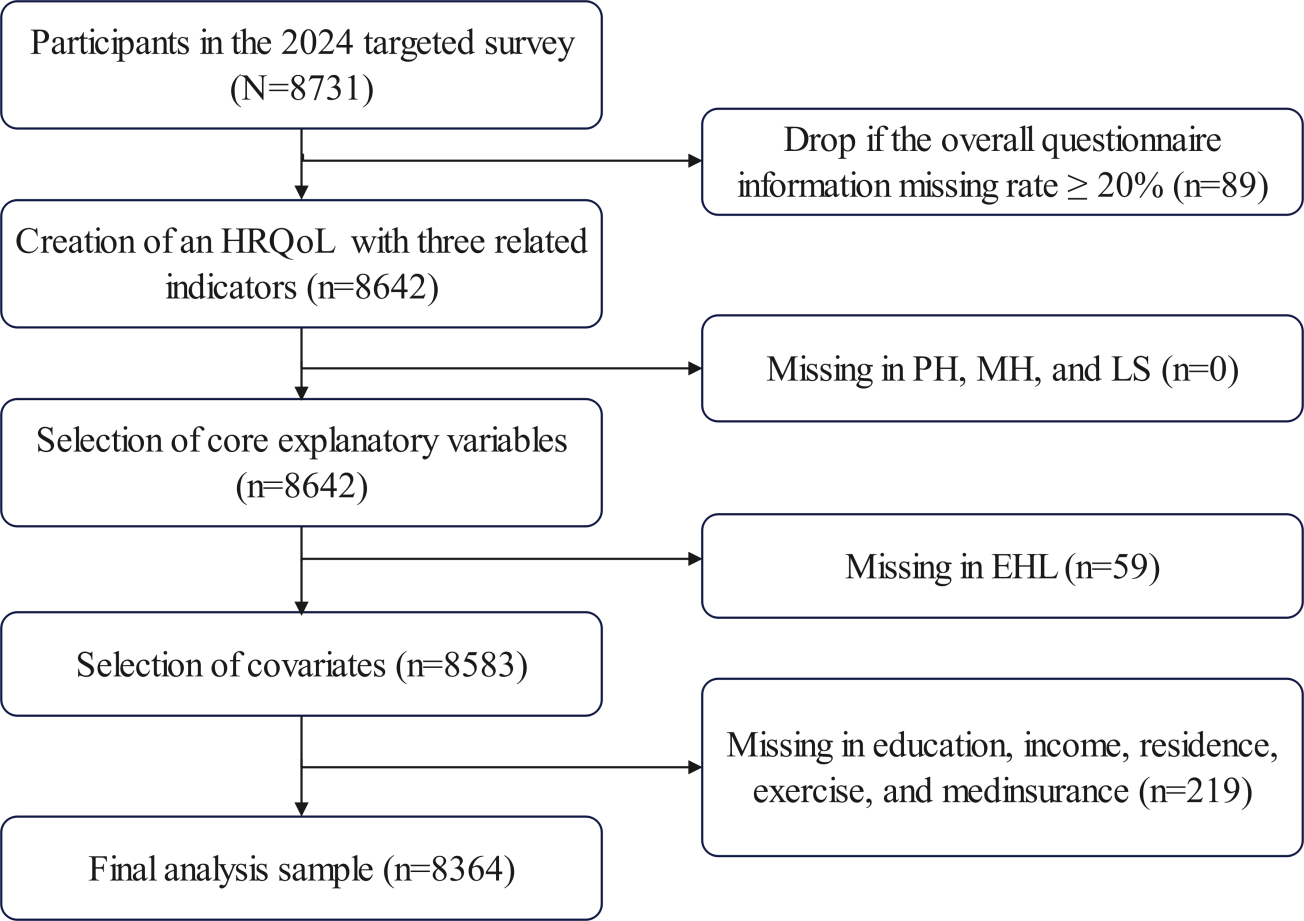
Flowchart showing the selection of participants. EHL: electronic health literacy; HRQoL: health-related quality of life; LS: life satisfaction; MH: mental health; PH: physical health.

A total of 8731 questionnaires were distributed to eligible individuals, resulting in a final valid response rate of 95.8%. Figure 1 illustrates the screening process for the study population.

### Measures

#### Assessment of HRQoL

The primary dependent variable was HRQoL. Quality of life is valued as a complement to conventional notions of health and functional status. An optimal health assessment should, therefore, encompass physical, social, and psychological functioning alongside quality of life [50]. Accordingly, this study assessed HRQoL using 3 indicators, namely, PH, MH, and LS.

PH was measured using the EuroQol Visual Analogue Scale (EQ-VAS), which is part of the EQ-5D instrument that evaluates individuals’ overall perception of their health status. Respondents rated their current health status on a visual scoring ruler ranging from 0 (“worst conceivable health state”) to 100 (“best conceivable health state”) [51]. The EQ-VAS is widely recognized as a straightforward, subjective instrument designed to capture general health status in clinical and population-based health studies [52].

MH was evaluated using the World Health Organization-Five Well-Being Index (WHO-5), which assesses subjective well-being and risk of depression. The WHO-5 questionnaire includes 5 specific measurement items, each rated on a 6-point Likert scale ranging from 0 (“at no time”) to 5 (“all of the time”). The raw total score, which spans from 0 to 25, is conventionally converted to a percentage scale by multiplying by 4, resulting in a range of 0 to 100. Higher values on this scale reflect a more favorable MH status [53,54]. In this study, the WHO-5 was found to have high internal consistency (Cronbach α=0.949; Kaiser-Meyer-Olkin [KMO]=.886).

LS is an individual’s overall assessment of their current life situation. It was measured with the Satisfaction With Life Scale (SWLS), developed by Diener et al [55]. As a classic and widely validated tool across populations and cultural contexts [56], the SWLS includes 5 items, each rated on a 7-point agreement scale. The summative score ranges from 5 to 35, and higher values mean better LS. The SWLS demonstrated high internal consistency in our sample (Cronbach α=0.961; KMO=.914).

#### Assessment of EHL

The core independent variable was EHL. It was measured using the 8-item eHealth Literacy Scale (eHEALS). Items are scored on a 5-point agreement scale (1=“strongly disagree,” 5=“strongly agree”), yielding a total from 8 to 40. Elevated scores correspond to a more positive self-assessment of one’s ability to obtain, judge, and use eHealth information [57]. In our study, the instrument showed excellent internal consistency (Cronbach α=0.986; KMO=.943). The scale has been widely applied in health behavior research across diverse populations and cultural settings [58].

#### Assessment of Mediating Variables

Two mediators included in this study were ATOA and SE. ATOA refers to an individual’s expectations, cognitive representations, and perceptions regarding their own aging process. It was measured by means of the Attitudes Toward One’s Own Aging subscale of the Philadelphia Geriatric Center Morale Scale. This subscale includes 5 items rated on a 5-point Likert scale (1=“strongly disagree,” 5=“strongly agree”), with 2 negative items reverse-coded. Average scores were calculated, with higher scores indicating more positive ATOA [59]. The internal consistency in this study was acceptable (Cronbach α=0.745; KMO=.811).

SE serves as a key internal motivator that drives individual behavior, influences emotional experiences, and ultimately shapes outcomes. SE was assessed using the General Self-Efficacy Scale (GSES), developed by Schwarzer and Jerusalem in 1995. The scale comprises 10 items rated on a 4-point Likert scale (1=“not at all true,” 4=“exactly true”). Average scores were computed, with higher scores reflecting stronger SE [60]. Reliability was excellent in our study (Cronbach α=0.965; KMO=.968).

#### Ascertainment of Covariates

To minimize potential confounding, this study incorporated a range of covariates based on prior literature [45,61]. These included demographic characteristics (age, gender, ethnicity, region, residence, education, and spouse status), socioeconomic factors (income, living arrangements, employment status, and number of children), lifestyle behaviors (caregiving for grandchildren, smoking, drinking, and exercise), social security status (participation in medical and pension insurance), and health-related variables (number of chronic diseases and use of health services). Detailed coding of variables is shown in Table 1.

**Table 1.** Variables encoding.

Variables	Definition	Type
Age	Actual age in years	Continuous
Sex	0=Female; 1=Male	Categorical
Ethnicity	0=Minority; 1=Han	Categorical
Region	1=Eastern; 2=Central; 3=Western	Categorical
Residence	0=Rural; 1=Urban	Categorical
Education	1=Below primary school; 2=Primary school; 3=Junior school; 4=Senior or vocational school; 5=Bachelor and above	Categorical
Spouse	0=Without; 1=With	Categorical
Income^a^	0=None; 1 =≤1000; 2=1001‐2000; 3=2001‐5000; 4 =≥5001	Categorical
Living	0=With family; 1=Empty nest	Categorical
Employment	0=Not; 1=Currently	Categorical
Children	0=None; 1=Have	Categorical
Grandcare	0=No; 1=Yes	Categorical
Smoking	0=No; 1=Yes	Categorical
Drinking	0=No; 1=Yes	Categorical
Exercise	0=No; 1=Yes	Categorical
Medical insurance	0=No; 1=Yes	Categorical
Pension insurance	0=No; 1=Yes	Categorical
Chronic	Actual number	Continuous
Outpatient	Actual frequency	Continuous
Hospitalization	Actual frequency	Continuous

a Income is denominated in Chinese yuan.

### Statistical Analysis

All statistical analyses were conducted using SPSS 25.0 (IBM Corp) and R 4.4.3 (R Foundation for Statistical Computing). Descriptive statistics, univariate analysis, and correlation analysis were performed. Continuous variables are reported as means and SDs, while categorical variables are reported as frequencies and percentages. To scrutinize the potential for common method variance, Harman single-factor test was performed. Given the correlations among the 3 healthy aging indicators, this study used seemingly unrelated regressions to simultaneously estimate the 3 equations, thereby improving estimation efficiency [62]. To control the increased familywise error rate due to multiple testing, the significance of the associations between EHL and the 3 HRQoL outcomes was assessed using a Bonferroni correction (adjusted *α*=.05/3). The mediation model, which specified ATOA and SE as parallel mediators, was tested with the PROCESS macro for SPSS (Model 4) [63]. To investigate the significance of the indirect effects, a bootstrapping procedure was applied based on 5000 resamples. Within each HRQoL dimension (PH, MH, and LS), the significance of indirect effects was evaluated using the Holm-Bonferroni sequential correction procedure. Heterogeneity analysis was conducted through subgroup regression. For these exploratory subgroup analyses, the false discovery rate (FDR) across all tests concerning the association between EHL and outcomes was controlled using the Benjamini-Hochberg procedure. All corrections pertained specifically to the hypothesis tests involving EHL. Statistical significance was tested using a 2-tailed test with an α level of .05, and the corrected significance level was fully considered.

### Ethical Considerations

This study was approved by the Ethics Committee of Zhongda Hospital, Southeast University (2024ZDSYLL294-Y01). All participants provided informed consent before their inclusion in the study. Anonymous data collection was used to ensure confidentiality, and no personal identifiers, such as names, were included in our study materials. No financial or material compensation was provided for participation in the study. The research strictly adhered to the principles of the Declaration of Helsinki (1964), the Council for International Organizations of Medical Sciences (CIOMS) International Ethical Guidelines, and the World Health Organization’s standards and procedures for research involving human participants.

## Results

### Descriptive Statistics

A total of 8364 participants were included in this study (3546 men and 4818 women), with a mean age of 70.83 (SD 8.39) years. In terms of residence, rural residents slightly outnumbered urban residents (56.5% vs 43.5%). Regarding education level, 31.3% (2614/8364) had not completed primary school, while only 3.9% (329/8364) held a bachelor’s degree or higher. In terms of lifestyle, the vast majority did not smoke (7036/8364, 84.1%) or drink alcohol (6743/8364, 80.6%). Regarding chronic disease prevalence, participants had an average of 1.26 types of chronic diseases (SD 1.14). The average EHL score was 20.13 (SD 10.58), indicating a relatively low level of EHL. The mean scores for PH, MH, and LS were 72.01 (SD 18.28), 71.18 (SD 20.47), and 26.71 (SD 5.52), respectively. Detailed participant characteristics are presented in Table 2.

**Table 2.** Descriptive statistics (N=8364).

Variables	Values
Age (years), mean (SD)	70.83 (8.39)
Sex, n (%)	
Female	4818 (57.6)
Male	3546 (42.4)
Ethnicity, n (%)	
Minority	1019 (12.2)
Han	7345 (87.8)
Region, n (%)	
Eastern	5259 (62.9)
Central	1670 (20.0)
Western	1435 (17.2)
Residence, n (%)	
Rural	4726 (56.5)
Urban	3638 (43.5)
Education, n (%)	
Below primary school	2614 (31.3)
Primary school	1422 (17.0)
Junior school	1979 (23.7)
Senior or vocational school	2020 (24.2)
Bachelor and above	329 (3.9)
Spouse, n (%)	
Without	1785 (21.3)
With	6579 (78.7)
Income^a^, n (%)	
None	1253 (15.0)
≤1000	2310 (27.6)
1001‐2000	1064 (12.7)
2001‐5000	2706 (32.4)
≥5001	1031 (12.3)
Living, n (%)	
With family	7207 (86.2)
Empty nest	1157 (13.8)
Employment, n (%)	
Not	6596 (78.9)
Currently	1768 (21.1)
Children, n (%)	
None	352 (4.2)
Have	8012 (95.8)
Grandcare, n (%)	
No	7089 (84.8)
Yes	1275 (15.2)
Smoking, n (%)	
No	7036 (84.1)
Yes	1328 (15.9)
Drinking, n (%)	
No	6743 (80.6)
Yes	1621 (19.4)
Exercise, n (%)	
No	3127 (37.4)
Yes	5237 (62.6)
Medical insurance, n (%)	
No	156 (1.9)
Yes	8208 (98.1)
Pension insurance, n (%)	
No	532 (6.4)
Yes	7832 (93.6)
Chronic, mean (SD)	1.26 (1.14)
Outpatient, mean (SD)	2.55 (4.82)
Hospitalization, mean (SD)	0.23 (0.67)

a Income is denominated in Chinese yuan.

### Common Method Bias Test

To assess the potential for common method bias, Harman single-factor test was performed on all measurement items. Six factors with eigenvalues greater than 1 were identified, with the first factor accounting for 38.76% of the variance—below the commonly accepted 50% threshold [64]. Consequently, these findings provide evidence that common method bias did not pose a significant threat to the validity of the study’s results.

### Univariate Analysis

Results of the univariate analysis are presented in Table 3. Significant differences in PH, MH, and LS were observed across region, residence, education, income, spouse, grandcare, and exercise (all *P*<.001). Overall, older adults living in eastern regions, with higher education and income levels, having a spouse, exercising regularly, or providing grandchild care exhibited better outcomes in healthy aging, whereas the effects of other factors were relatively weaker.

**Table 3.** Univariate analysis results.

Variables	PH^a^			MH^b^			LS^c^		
	*t* test (*df*)	*F* test (*df*)	*P* value	*t* test (*df*)	*F* test (*df*)	*P* value	*t* test (*df*)	*F* test (*df*)	*P* value
Sex	−0.345 (8362)	—^d^	.73	0.791 (8362)	—	.43	2.736 (8362)	—	.006
Ethnicity	−0.543 (8362)	—	.59	−6.845 (8362)	—	<.001	−6.392 (8362)	—	<.001
Region	—	152.358 (2, 8361)	<.001	—	242.853 (2, 8361)	<.001	—	265.219 (2, 8361)	<.001
Residence	6.003 (8362)	—	<.001	−6.136 (8362)	—	<.001	3.661 (8362)	—	<.001
Education	—	18.259 (4, 8359)	<.001	—	42.114 (4, 8359)	<.001	—	21.243 (4, 8359)	<.001
Spouse	−4.252 (8362)	—	<.001	−5.838 (8362)	—	<.001	−6.546 (8362)	—	<.001
Income	—	17.465 (4, 8359)	<.001	—	39.202 (4, 8359)	<.001	—	48.025 (4, 8359)	<.001
Living	1.248 (8362)	—	.21	2.326 (8362)	—	.02	2.588 (8362)	—	.01
Employment	−1.261 (8362)	—	.21	3.691 (8362)	—	<.001	0.854 (8362)	—	.39
Children	6.070 (8362)	—	<.001	−0.484 (8362)	—	.63	−1.143 (8362)	—	.25
Grandcare	−8.881 (8362)	—	<.001	−6.695 (8362)	—	<.001	−5.350 (8362)	—	<.001
Smoking	−0.561 (8362)	—	.58	3.715 (8362)	—	<.001	3.752 (8362)	—	<.001
Drinking	−1.350 (8362)	—	.18	−0.764 (8362)	—	.45	4.253 (8362)	—	<.001
Exercise	−8.346 (8362)	—	<.001	−15.050 (8362)	—	<.001	−11.874 (8362)	—	<.001
Medical insurance	−0.797 (8362)	—	.43	−2.545 (8362)	—	.01	−4.622 (8362)	—	<.001
Pension insurance	1.026 (8362)	—	.31	−1.204 (8362)	—	.23	−0.751 (8362)	—	.45

a PH: physical health.

b MH: mental health.

c LS: life satisfaction.

d Not applicable.

### Correlation Analysis

**Figure 2.**
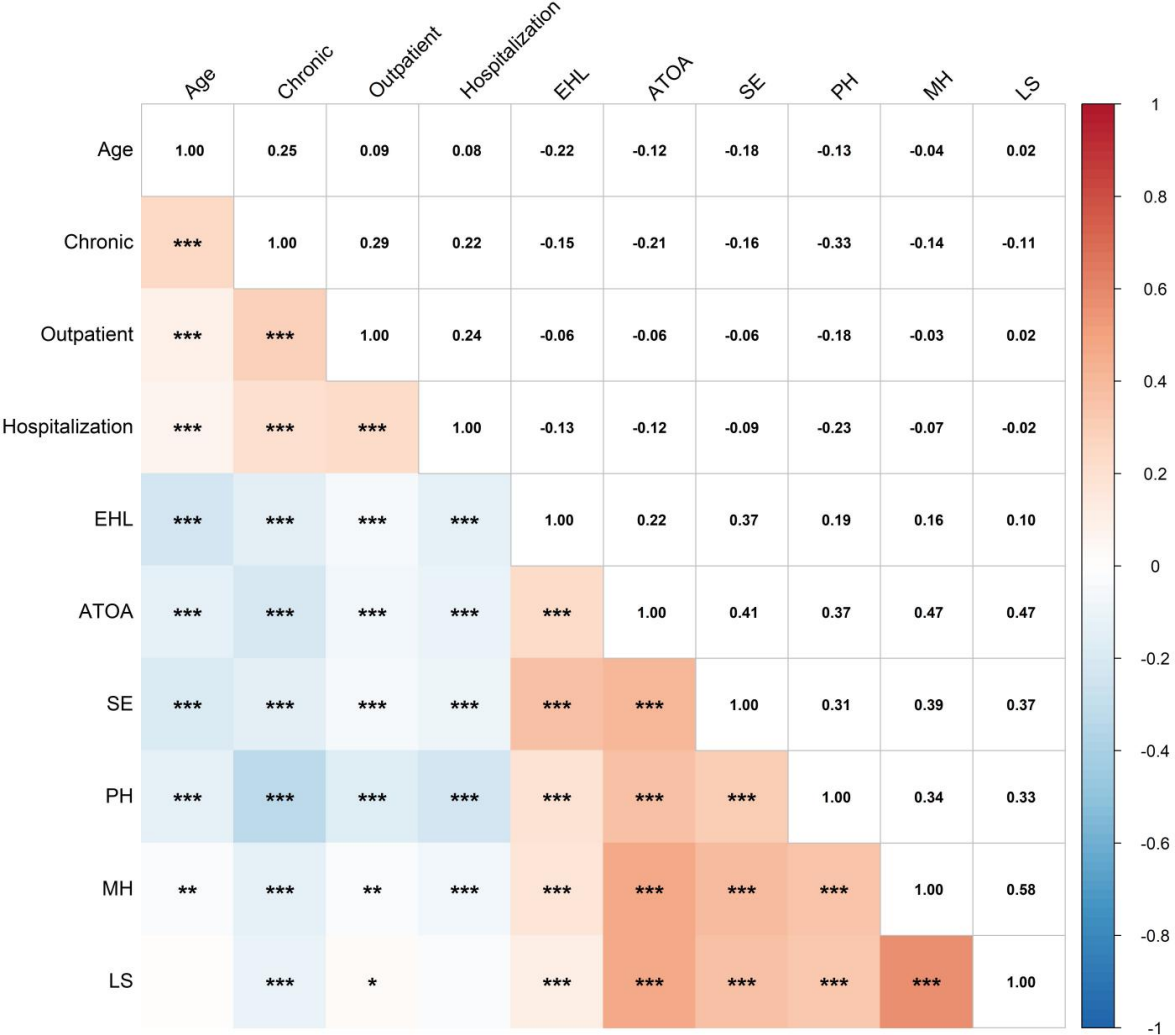
Correlation heatmap. *P<.05; **P<.01; ***P<.001. ATOA: attitudes toward own aging; EHL: electronic health literacy; LS: life satisfaction; MH: mental health; PH: physical health; SE: self-efficacy.

Given that our study includes multiple variable types and some continuous variables deviate from the normal distribution, Spearman correlation analysis was used to assess the associations among age, chronic, outpatient, hospitalization, EHL, ATOA, SE, PH, MH, and LS. Results are shown in Figure 2. EHL was positively correlated with ATOA, SE, and HRQoL (=0.102‐0.370; *P*<.05). In contrast, aside from age and hospitalizations, the number of chronic diseases and outpatient visits was negatively correlated with EHL, ATOA, SE, and HRQoL (= –0.331 to 0.030; *P*<.05).

### Basic Regression

Based on the univariate analysis results, seemingly unrelated regressions were used to simultaneously estimate PH, MH, and LS (Table 4). In Model 1, EHL was positively associated with PH, MH, and LS (all *P*<.001). After controlling for covariates in Model 2, the positive associations between EHL and the 3 outcomes remained significant (all *P*<.001). Notably, these associations retained their statistical significance even after applying a Bonferroni correction for the 3 comparisons (adjusted *α*=0.0167). These findings confirm that EHL exerts a robust positive influence on multiple dimensions of HRQoL, thereby supporting hypothesis 1.

**Table 4.** Baseline regression results.^a^

Variables	Model 1^b^			Model 2^c^		
	PH^d^	MH^e^	LS^f^	PH	MH	LS
EHL^g^	0.235^h^ (0.018)	0.273^h^ (0.021)	0.064^h^ (0.006)	0.273^h^ (0.189)	0.190^h^ (0.026)	0.082^h^ (0.007)
Age	—^i^	—	—	0.147 (0.025)	0.085^j^ (0.030)	0.053^h^ (0.008)
Ethnicity	—	—	—	−6.734^h^ (0.630)	−0.756 (0.811)	−0.902^h^ (0.197)
Region						
Central				−7.180^h^ (0.501)	−7.655^h^ (0.642)	−2.741^h^ (0.167)
Western				−15.127^h^ (0.644)	−10.843^h^ (0.754)	−3.639^h^ (0.199)
Residence				−6.761^h^ (0.656)	−4.701^h^ (0.750)	−3.639^h^ (0.202)
Education						
Primary school				−0.399 (0.519)	−1.018 (0.442)	−0.212 (0.170)
Junior school				1.306^k^ (0.550)	0.442 (0.675)	0.042 (0.179)
Senior or vocational school				4.399^h^ (0.686)	3.041^h^ (0.788)	0.922^h^ (0.206)
Bachelor and above				4.296^h^ (1.098)	4.097^j^ (1.321)	1.203^j^ (0.348)
Spouse				0.261 (0.624)	2.293^j^ (0.762)	0.874^h^ (0.192)
Income						
≤1000				0.613 (0.517)	1.929^j^ (0.710)	2.074^h^ (0.179)
1001–2000				1.888^j^ (0.629)	2.372^j^ (0.785)	1.450^h^ (0.207)
2001–5000				1.723^k^ (0.756)	6.774^h^ (0.917)	2.034^h^ (0.235)
≥5001				2.396^j^ (0.832)	5.383^h^ (1.085)	2.609^h^ (0.285)
Living				0.515 (0.693)	0.406 (0.871)	0.035 (0.226)
Grandcare				1.468^j^ (0.446)	2.225^h^ (0.573)	0.268 (0.155)
Smoking				−0.192 (0.475)	−1.611^j^ (0.626)	−0.586^h^ (0.158)
Exercise				2.078^h^ (0.397)	4.939^h^ (0.483)	1.064^h^ (0.125)
Medical insurance				1.198 (1.332)	4.118^k^ (1.688)	2.059^h^ (0.485)
Chronic				−2.903^h^ (0.174)	−2.027^h^ (0.207)	−0.569^h^ (0.056)
Outpatient				−0.290^h^ (0.036)	−0.078 (0.042)	−0.012 (0.012)
Hospitalization				−4.188^h^ (0.312)	−1.259^h^ (0.344)	−0.256^j^ (0.097)
Observations	8364	8364	8364	8364	8364	8364
*R* ^2^	0.019	0.02	0.015	0.19	0.116	0.155

a All categorical variables are coded with the first group as the reference category. Robust standard errors are shown in parentheses. The significance of the association between EHL and HRQoL dimensions was corrected using the Bonferroni method (corrected α=0.0167), with all *P*_Bonf _<.05.

b Model 1: Unadjusted.

c Model 2: Adjusted for age, ethnicity, region, residence, education, spouse, income, living, grandcare, smoking, exercise, medical insurance, chronic, outpatient, and hospitalization.

d PH: physical health.

e MH: mental health.

f LS: life satisfaction.

g EHL: electronic health literacy.

h *P*<.001.

i Not applicable.

j *P*<.01.

k *P*<.05.

### Mediation Analysis

Mediation analysis was undertaken using PROCESS Model 4 in SPSS, with ATOA and SE specified as parallel mediators. After controlling for covariates, the model included EHL as the predictor, ATOA and SE as mediators, and PH, MH, and LS as outcomes (Table S1 in Multimedia Appendix 1). Results showed that EHL positively predicted both ATOA (*β*=0.015; *P*<.001) and SE (*β*=0.019; *P*<.001), indicating that higher EHL was associated with more positive aging attitudes and stronger self-efficacy.

For PH, EHL maintained a significant positive association (*β*=0.128; *P*<.001), and both ATOA (*β*=3.270; *P*<.001) and SE (*β*=5.129; *P*<.001) strongly predicted PH. Thus, EHL directly promoted PH and also indirectly contributed via ATOA and SE. For MH, the direct association between EHL and MH became negative after including the mediators (*β*=–.069; *P*<.01). However, both ATOA (*β*=9.417; *P*<.001) and SE (*β*=6.929; *P*<.001) exerted strong positive effects on MH, suggesting that the positive relationship between EHL and MH was almost entirely mediated by ATOA and SE, with evidence of a suppression effect. For LS, the direct effect of EHL was nonsignificant, while both ATOA (*β*=2.583; *P*<.001) and SE (*β*=2.250; *P*<.001) positively predicted LS. This indicates that the association between EHL and LS was fully mediated by ATOA and SE.

**Figure 3.**
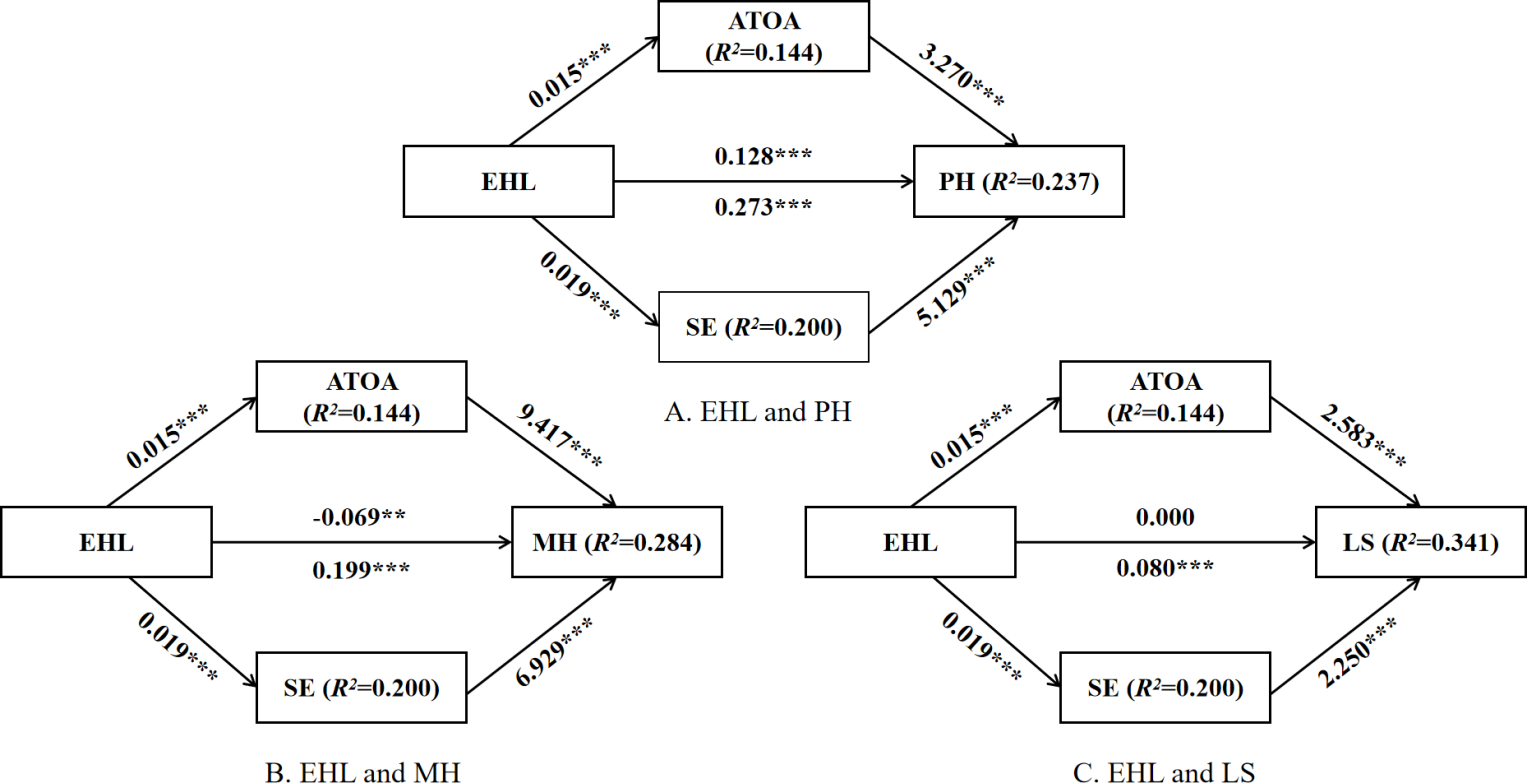
Mediation model. **P<.01; ***P<.001. ATOA: attitudes toward own aging; EHL: electronic health literacy; LS: life satisfaction; MH: mental health; PH: physical health; SE: self-efficacy.

Following established recommendations [65], bootstrapping with 5000 resamples was used to assess mediation significance via 95% CI (Table 5; Figure 3). For PH, the direct effect of EHL was significant (95% CI 0.086-0.170), and the indirect effects of ATOA (95% CI 0.038-0.059) and SE (95% CI 0.081-0.112) were also significant, with total indirect effects accounting for 52.7% of the total effect. For MH, the direct effect of EHL was negative and significant (95% CI –0.115 to –0.024), but the indirect effects through ATOA (95% CI 0.119-0.158) and SE (95% CI 0.112-0.149) were positive, yielding a positive total effect (95% CI 0.151-0.248)—indicating inconsistent mediation. For LS, the direct effect of EHL was nonsignificant (95% CI –0.012 to 0.012), while the indirect effects of ATOA (95% CI 0.033-0.043) and SE (95% CI 0.036-0.047) were significant, with total indirect effects accounting for 100% of the total effect. Furthermore, after using Holm-Bonferroni sequential correction, it was found that the bootstrap CI for all indirect effects did not include zero, indicating that all mediating paths remained statistically significant. These results support hypotheses 2a and 2b.

**Table 5.** Significance test for mediating effect^a^.

Variables	Effect	Paths	β (SE)	95% CI	Proportion
PH^b^	Direct	EHL^c^ →PH	.128 (0.022)	[0.086-0.170]	45.8%
	Indirect	EHL→ATOA^d^ →PH	.048 (0.005)	[0.038-0.059]	17.6%
		EHL→SE^e^→PH	.096 (0.008)	[0.081-0.112]	35.2%
		Total Indirect	.144 (0.009)	[0.126-0.163]	52.7%
	Total		.273 (0.021)	[0.231-0.314]	—^f^
MH^g^	Direct	EHL→MH	–.069 (0.023)	[–0.115 to –0.024]	—
	Indirect	EHL→ATOA→MH	.138 (0.010)	[0.119-0.158]	—
		EHL→SE→MH	.130 (0.010)	[0.112-0.149]	—
		Total Indirect	.268 (0.014)	[0.240-0.296]	—
	Total		.199 (0.025)	[0.151-0.248]	—
LS^h^	Direct	EHL→LS	.000 (0.006)	[–0.012 to 0.012]	0.0%
	Indirect	EHL→ATOA^d^→LS	.038 (0.003)	[0.033-0.043]	47.5%
		EHL→SE→LS	.042 (0.003)	[0.036-0.047]	52.5%
		Total Indirect	.080 (0.004)	[0.071-0.088]	100%
	Total		0.080 (0.007)	[0.067-0.093]	—

a Proportion is the effect size ratio; for mental health, direct and total effects have opposite signs (inconsistent mediation), so proportion is not reported. After applying the Holm-Bonferroni sequential correction procedure, the bootstrap CI for all indirect effects did not include zero.

b PH: physical health.

c EHL: electronic health literacy.

d ATOA: attitudes toward own aging.

e SE: self-efficacy.

f Not applicable.

g MH: mental health.

h LS: life satisfaction.

### Heterogeneity Test

Our study further conducted subgroup analyses stratified by age, residence, and physical activity behavior, and the results are presented in Tables S2-S4 in Multimedia Appendix 1. Given that subgroup analyses involve multiple comparisons, we performed FDR correction (Benjamini-Hochberg) for all association tests between EHL and different HRQoL dimensions across all subgroups. All significant associations remained significant after correction (all *P*_BH-FDR_<.05), indicating that the subgroup analysis results are robust.

The role of EHL showed notable age differences. Among the younger-old adults (≤74 years), EHL had the strongest association with PH (*β*=0.288; *P*<.001), and also demonstrated significant positive effects on MH (*β*=0.188; *P*<.001) and LS (*β*=0.089*; P*<.001). This indicates that, within this group, EHL could more comprehensively enhance their HRQoL. In contrast, among the older-old adults (>74 years), EHL still had significant positive predictive effects on all 3 dimensions, but its strength was generally weaker. The coefficient for PH decreased to  0.222 (*P*<.001), and the promoting effects on MH (*β*=0.175; *P*<.001) and LS (*β*=0.060; *P*<.001) were also lower than those in the younger group. These results support hypothesis 3a.

Significant urban-rural differences were observed. Among rural older adults, EHL was significantly and positively associated with PH (*β*=0.215; *P*<.001), but its association with MH was nonsignificant, and its effect on LS was positive but relatively weak (*β*=.019; *P*=.032). In contrast, among urban older adults, EHL was more strongly associated with PH (*β*=0.237; *P*<.001), and its associations with MH and LS were also considerably stronger than in rural areas. Overall, EHL had a broader impact on HRQoL in urban older adults, while in rural populations, its health value was concentrated primarily in PH. These findings support hypothesis 3b.

Associations also show differences with varying exercise habits. Among nonexercisers, EHL had its strongest positive association with PH (*β*=0.322; *P*<.001), suggesting that EHL effectively compensated for health deficits due to lack of exercise and served as a core support for improving PH. EHL also positively predicted MH (*β*=0.196; *P*<.001) and LS (*β*=0.067*;P*<.001) in this group. In contrast, among exercisers, the health effects of EHL, though still significant, were weaker. The association with PH dropped to *β*=0.193 (*P*<.001), while its effect on MH (*β*=0.147; *P*<.001) was also lower than in nonexercisers, and the effect on LS showed little difference. These findings suggest that nonexercisers’ HRQoL was more strongly dependent on EHL, whereas exercisers already benefited from the baseline health improvements of physical activity, limiting the additional value of EHL. These results support hypothesis 3c.

## Discussion

### Principal Findings

This study found that EHL is significantly associated with HRQoL among older adults in China. The enhancement of EHL contributes to improving HRQoL in older adults. Previous studies have established a positive association between general health literacy and HRQoL [66], while relevant systematic reviews have clarified the intrinsic relationship between EHL and multidimensional health outcomes [25]. For Chinese older adults, a prior study conducted in Jinan City initially explored the relationship between EHL and HRQoL [45]. These findings are consistent with our conclusions. Our study further expands the representativeness of the sample and the generalizability of conclusions, providing empirical evidence for extending research in this field to broader populations of Chinese older adults.

The mechanism of EHL varies across different dimensions of HRQoL. EHL exerted a direct effect on PH, whereas its associations with MH and LS were almost entirely mediated by ATOA and SE. Older adults with higher EHL can more efficiently retrieve, comprehend, and apply online health information, enabling more scientifically grounded disease prevention, symptom management, medication adherence, and health care decision-making [67]. This process involves a direct conversion of cognitive and behavioral skills, yielding immediate, instrumental positive effects on PH. This direct effect fundamentally reflects the immediacy of translating information literacy into health practices, highlighting EHL’s core value as a capacity for health information use [68].

An inconsistent mediation was observed in the MH model. The direct effect of EHL on MH was negative, yet the indirect effects via ATOA and SE were positive, resulting in a positive total effect. Possessing high EHL may also expose individuals to an overwhelming volume of health information, potentially triggering cognitive overload and uncertainty [69]. This fosters negative emotions such as health anxiety and cyberhypochondria. Catastrophizing minor symptoms further exacerbates psychological burden [70]. Older adults may intensify perceptions of health limitations or experience frustration from digital operational barriers, amplifying direct negative impacts. However, EHL can exert protective effects by fostering positive psychological mechanisms, as ATOA reduces age-related health fears, while SE enhances confidence in tackling health challenges [71,72]. Both collectively boost psychological resilience and emotional regulation capacity. This indicates that benefits to older adults’ MH stem not from the information itself, but from the psychological coping mechanisms constructed through EHL. Without such transformative processes, high EHL may instead pose risks to the psychological well-being of older adults.

For LS, the association between EHL and outcomes was fully mediated by ATOA and SE. As a cognitive evaluation of overall life quality, LS is more closely associated with universal psychological resources and meaning frameworks [73]. EHL, as a tool-based skill, does not directly contribute to overall life evaluations. Instead, EHL is more likely to enhance LS by strengthening positive aging attitudes (enabling older adults to embrace the life course) and leveraging SE to implement health-promoting behaviors. In other words, EHL functions as an enabler: the knowledge and sense of control afforded by EHL are filtered and sublimated through an individual’s meaning system and core beliefs before being translated into more holistic LS.

The analysis also revealed heterogeneity. The health benefits gained by the younger-old from EHL are the most comprehensive and pronounced, indicating that their physiological and cognitive reserves can more effectively translate health literacy into well-being. The intensity of benefits for the older-old adults generally diminishes, potentially stemming from the fact that the marginal benefits of EHL may decrease with age due to cumulative health deficits and the dominance of aging processes [74]. Urban older adults benefited more comprehensively from EHL, likely due to stronger resource conditions that facilitated the translation of literacy into gains in MH and LS, while among rural older adults, the benefits of EHL were concentrated on PH [75]. Similarly, nonexercisers derived greater benefits from EHL, suggesting that it compensates for health deficits due to physical inactivity [76]. Among exercisers, the marginal effects of EHL were weaker, as they already benefited from baseline advantages of physical activity.

### Contributions and Implications

This study makes 3 key contributions. First, it extends the literature by showing that EHL is linked not only to PH but also to MH and LS. Second, it identifies ATOA and SE as psychosocial mechanisms through which EHL influences HRQoL. Third, it reveals heterogeneity across subgroups, highlighting the compensatory role of EHL for vulnerable populations such as rural residents and nonexercisers.

These findings confirm the necessity of integrating EHL into healthy aging policies. Policymakers should treat EHL as a social determinant of health and incorporate it into digital inclusion and aging strategies, with particular attention to disadvantaged groups. Practical approaches include (1) recruiting family doctors and volunteers to form a silver-haired lecturer team, implementing EHL training programs at the community level, and establishing personalized advancement pathways for literacy development. The mature models of Shanghai’s “Senior Digital Life Workshops” and “Senior Health Cloud Classroom” can serve as references. (2) Rooted in China’s unique filial piety and integrity culture, advocating intergenerational interaction models featuring digital reciprocity, mutual learning, and generational integration. (3) With reference to the practical experience of aging-friendly digital infrastructure construction in provinces such as Jiangsu, Zhejiang, and Guangdong, nationwide standardized upgrades and renovations of age-friendly digital infrastructure should be promoted. (4) Expanding digital health services and digital life services that are compatible with the literacy level of the older adults, such as disease prevention and management, health promotion, MH care, and medical electronic commerce, to effectively meet the needs of the older adults. Overall, the comprehensive application of strategies such as community training, intergenerational teaching, infrastructure upgrading projects, and inclusive service expansion can enhance older adults’ confidence in using digital health resources, foster a more positive attitude toward aging, strengthen SE, and ultimately improve HRQoL. In addition, EHL should be incorporated into the policy evaluation framework. For example, in the policy evaluation of advancing the informatization construction of compact county medical communities, indicators such as the improvement rate of EHL among the older adults in the county and the use rate of digital health tools among rural older adults should be included in the evaluation system. This measure can align digital health initiatives with the public health goals of health equity and digital inclusion, ensuring that the development of digital health will not widen existing health gaps but instead serve as a catalyst for promoting universal healthy aging in the digital era.

### Limitations

Inevitably, our study has several limitations. First, the cross-sectional design precludes causal inference; longitudinal follow-ups are needed. Second, although the sampling ensured regional and population representativeness, generalizability to other cultural contexts remains limited. Third, while common method bias was tested and found to be minimal, the influence of unmeasured confounding cannot be completely excluded. For instance, our study did not account for confounding factors among older adults. Finally, our study treats ATOA and SE as parallel mediating variables. Future research should explore the potential dynamic interdependence and interactions between them to deepen our understanding of the mediating mechanism.

### Conclusions

In conclusion, EHL is strongly associated with HRQoL among older adults. Its association with PH appears relatively direct, whereas its associations with MH and LS are largely mediated by ATOA and SE. The observed heterogeneity by age, residence, and exercise behavior suggests that targeted interventions are needed for specific subgroups. Overall, this study unveils the potential of EHL as a key lever for promoting healthy aging in the digital era, emphasizing the urgency of bridging the digital divide and cultivating EHL to support the well-being of older adults.

## Supplementary material

10.2196/84700 Multimedia Appendix 1 Mediation analysis and heterogeneity analysis results.

## Abbreviations

ATOA: attitudes toward own aging
CIOMS: Council for International Organizations of Medical Sciences
eHEALS: eHealth Literacy Scale
EHL: electronic health literacy
EQ-VAS: EuroQol Visual Analogue Scale
FDR: false discovery rate
GSES: General Self-Efficacy Scale
HRQoL: health-related quality of life
KMO: Kaiser-Meyer-Olkin
LS: life satisfaction
MH: mental health
PH: physical health
SE: self-efficacy
SWLS: Satisfaction With Life Scale
WHO-5: World Health Organization-Five Well-Being Index

## References

[1] Christensen K, Doblhammer G, Rau R, Vaupel JW (2009). Ageing populations: the challenges ahead. Lancet.

[2] Beard JR, Officer A, de Carvalho IA (2016). The world report on ageing and health: a policy framework for healthy ageing. Lancet.

[3] Wang HM, Beyer M, Gensichen J, Gerlach FM (2008). Health-related quality of life among general practice patients with differing chronic diseases in Germany: cross sectional survey. BMC Public Health.

[4] Chen X, Giles J, Yao Y (2022). The path to healthy ageing in China: a Peking University-Lancet Commission. Lancet.

[5] Chen C, Liu GG, Shi QL (2020). Health-related quality of life and associated factors among oldest-old in China. J Nutr Health Aging.

[6] Li H, Tao S, Sun S, Xiao Y, Liu Y (2024). The relationship between health literacy and health-related quality of life in Chinese older adults: a cross-sectional study. Front Public Health.

[7] Moorhead SA, Hazlett DE, Harrison L, Carroll JK, Irwin A, Hoving C (2013). A new dimension of health care: systematic review of the uses, benefits, and limitations of social media for health communication. J Med Internet Res.

[8] Hunsaker A, Hargittai E (2018). A review of Internet use among older adults. New Media Soc.

[9] Mitchell UA, Chebli PG, Ruggiero L, Muramatsu N (2019). The digital divide in health-related technology use: the significance of race/ethnicity. Gerontologist.

[10] Kruse C, Fohn J, Wilson N, Nunez Patlan E, Zipp S, Mileski M (2020). Utilization barriers and medical outcomes commensurate with the use of telehealth among older adults: systematic review. JMIR Med Inform.

[11] Norman CD, Skinner HA (2006). eHealth literacy: essential skills for consumer health in a networked world. J Med Internet Res.

[12] Çöme O, Gökdemir Ö, Bilik Sezer B, Kasapoğlu SS, Kjær NK, Güldal D (2025). The interplay between cognitive function and digital health literacy among older adults: implications for e-health equity and accessibility. Int J Med Inform.

[13] Wu Y, Wen J, Wang X (2022). Associations between e-health literacy and chronic disease self-management in older Chinese patients with chronic non-communicable diseases: a mediation analysis. BMC Public Health.

[14] Ivan L, Marston HR, Prabhu VG (2024). Successful aging across middle versus high-income countries: an analysis of the role of eHealth literacy associated with loneliness and well-being. Gerontologist.

[15] Chen A, Wang X, Xu A, Liu Q, Tong B (2025). Analysis of the impact of e-health literacy on health behavior among the elderly in Jiangsu: structural equation model. Public Health Nurs.

[16] Xie L, Mo PKH (2024). A 3-wave longitudinal study of eHealth literacy and older people’s health-related quality of life in China: the mediating role of general self-efficacy. J Am Med Dir Assoc.

[17] Stellefson M, Paige SR, Alber JM (2019). Association between health literacy, electronic health literacy, disease-specific knowledge, and health-related quality of life among adults with chronic obstructive pulmonary disease: cross-sectional study. J Med Internet Res.

[18] Zhang M, Tao S, Ge X (2025). Health self-management behaviors as a bridge between electronic health literacy and health-related quality of life: cross-sectional study from China. J Med Internet Res.

[19] Lai Y, Chen S, Li M, Ung COL, Hu H (2021). Policy interventions, development trends, and service innovations of internet hospitals in China: documentary analysis and qualitative interview study. J Med Internet Res.

[20] Jokisch MR, Schmidt LI, Doh M (2022). Acceptance of digital health services among older adults: findings on perceived usefulness, self-efficacy, privacy concerns, ICT knowledge, and support seeking. Front Public Health.

[21] (2020). Decade of healthy ageing: baseline report. https://apps.who.int/iris/handle/10665/338677.

[22] Singh S, Goodwin S, Zhong S (2024). Inequalities in health-related quality of life and functional health of an aging population: a Canadian community perspective. PLoS One.

[23] Tan G, Chao J, Jin S (2025). The association between digital health literacy and health inequalities among Chinese older adults: a multicenter cross-sectional study. Digit Health.

[24] van Kessel R, Wong BLH, Clemens T, Brand H (2022). Digital health literacy as a super determinant of health: more than simply the sum of its parts. Internet Interv.

[25] Xie L, Zhang S, Xin M, Zhu M, Lu W, Mo PKH (2022). Electronic health literacy and health-related outcomes among older adults: a systematic review. Prev Med.

[26] Rowe JW, Kahn RL (1997). Successful aging. Gerontologist.

[27] Levy BR, Slade MD, Kasl SV (2002). Longitudinal benefit of positive self-perceptions of aging on functional health. J Gerontol B Psychol Sci Soc Sci.

[28] Di X, Wang L (2025). Impact of digital literacy on aging attitudes of the elderly from the perspective of social cognitive theory. BMC Public Health.

[29] Bellingtier JA, Neupert SD (2018). Negative aging attitudes predict greater reactivity to daily stressors in older adults. J Gerontol B Psychol Sci Soc Sci.

[30] Levy BR, Myers LM (2004). Preventive health behaviors influenced by self-perceptions of aging. Prev Med.

[31] Levy BR, Hausdorff JM, Hencke R, Wei JY (2000). Reducing cardiovascular stress with positive self-stereotypes of aging. J Gerontol B Psychol Sci Soc Sci.

[32] Paasche-Orlow MK, Wolf MS (2007). The causal pathways linking health literacy to health outcomes. Am J Health Behav.

[33] Bandura A (1997). Self-Efficacy: The Exercise of Control.

[34] Park C (2025). Electronic health literacy as a source of self-efficacy among community-dwelling older adults. Clin Gerontol.

[35] Bandura A (1977). Self-efficacy: toward a unifying theory of behavioral change. Psychol Rev.

[36] Bandura A (2004). Health promotion by social cognitive means. Health Educ Behav.

[37] Du S, Tian L, Tian Y, Feng Z, Wang Y (2023). The role of self-efficacy and self-care agency as mediating factors in the link between health literacy and health-promoting lifestyle among older adults post covid 19 era: a multiple mediator model. Geriatr Nurs.

[38] Arcaya MC, Arcaya AL, Subramanian SV (2015). Inequalities in health: definitions, concepts, and theories. Glob Health Action.

[39] Jiang X, Wang L, Leng Y (2024). The level of electronic health literacy among older adults: a systematic review and meta-analysis. Arch Public Health.

[40] Deng SY, Zhao IY, Ho MH, Saravanakumar P, Molassiotis A, Montayre J (2022). Rural-urban disparities in healthy ageing: evidence from a national study in China. Collegian.

[41] Pailaha AD (2023). Public health nursing: Challenges and innovations for health literacy in rural area. Public Health Nurs.

[42] Baltes PB, Baltes MM, Baltes PB, Baltes MM (1990). Successful Aging: Perspectives from the Behavioral Sciences European Network on Longitudinal Studies on Individual Development.

[43] Zheng X, Xue Y, Dong F (2022). The association between health-promoting-lifestyles, and socioeconomic, family relationships, social support, health-related quality of life among older adults in China: a cross sectional study. Health Qual Life Outcomes.

[44] Cui K, Zou W, Ji X, Zhang X (2024). Does digital technology make people healthier: the impact of digital use on the lifestyle of Chinese older adults. BMC Geriatr.

[45] Li S, Cui G, Yin Y, Wang S, Liu X, Chen L (2021). Health-promoting behaviors mediate the relationship between eHealth literacy and health-related quality of life among Chinese older adults: a cross-sectional study. Qual Life Res.

[46] Lin CY, Ganji M, Griffiths MD, Bravell ME, Broström A, Pakpour AH (2020). Mediated effects of insomnia, psychological distress and medication adherence in the association of eHealth literacy and cardiac events among Iranian older patients with heart failure: a longitudinal study. Eur J Cardiovasc Nurs.

[47] Leung AYM, Parial LL, Tolabing MC (2022). Sense of coherence mediates the relationship between digital health literacy and anxiety about the future in aging population during the COVID-19 pandemic: a path analysis. Aging Ment Health.

[48] Choi M (2020). Association of eHealth use, literacy, informational social support, and health-promoting behaviors: mediation of health self-efficacy. Int J Environ Res Public Health.

[49] Neter E, Brainin E (2019). Association between health literacy, eHealth literacy, and health outcomes among patients with long-term conditions. Eur Psychol.

[50] The Whoqol Group (1998). The World Health Organization quality of life assessment (WHOQOL): development and general psychometric properties. Soc Sci Med.

[51] Cheng LJ, Tan RLY, Luo N (2021). Measurement properties of the EQ VAS around the globe: a systematic review and meta-regression analysis. Value Health.

[52] Hu W, Zhou L, Chu J (2022). Estimating population norms for the health-related quality of life of adults in southern Jiangsu Province, China. Sci Rep.

[53] (2024). The World Health Organization-five well-being index (WHO-5). World Health Organization.

[54] Topp CW, Østergaard SD, Søndergaard S, Bech P (2015). The WHO-5 Well-Being Index: a systematic review of the literature. Psychother Psychosom.

[55] Diener E, Emmons RA, Larsen RJ, Griffin S (1985). The Satisfaction With Life Scale. J Pers Assess.

[56] Whisman MA, Judd CM (2016). A cross-national analysis of measurement invariance of the Satisfaction With Life Scale. Psychol Assess.

[57] Norman CD, Skinner HA (2006). eHEALS: the eHealth Literacy Scale. J Med Internet Res.

[58] Oh SS, Kim KA, Kim M, Oh J, Chu SH, Choi J (2021). Measurement of digital literacy among older adults: systematic review. J Med Internet Res.

[59] Neupert SD, Bellingtier JA (2017). Aging attitudes and daily awareness of age-related change interact to predict negative affect. Gerontologist.

[60] Scholz U, Doña BG, Sud S (2002). Is general self-efficacy a universal construct? Psychometric findings from 25 countries. Eur J Psychol Assess.

[61] Liu S, Lu Y, Wang D (2023). Impact of digital health literacy on health-related quality of life in Chinese community-dwelling older adults: the mediating effect of health-promoting lifestyle. Front Public Health.

[62] Chai Y, Xian G, Wang M, Guo L, Luo S (2024). Aging wisely: the impact of Internet use on older adults’ mental health. J Affect Disord.

[63] Preacher KJ, Hayes AF (2004). SPSS and SAS procedures for estimating indirect effects in simple mediation models. Behav Res Methods Instrum Comput.

[64] Podsakoff PM, MacKenzie SB, Lee JY, Podsakoff NP (2003). Common method biases in behavioral research: a critical review of the literature and recommended remedies. J Appl Psychol.

[65] Hayes AF (2013). Introduction to Mediation, Moderation, and Conditional Process Analysis: A Regression-Based Approach.

[66] Panagioti M, Skevington SM, Hann M (2018). Effect of health literacy on the quality of life of older patients with long-term conditions: a large cohort study in UK general practice. Qual Life Res.

[67] Lee EH, Lee YW, Kang EH, Kang HJ (2024). Relationship between electronic health literacy and self-management in people with type 2 diabetes using a structural equation modeling approach. J Nurs Res.

[68] Huhta AM, Hirvonen N, Huotari ML (2018). Health literacy in web-based health information environments: systematic review of concepts, definitions, and operationalization for measurement. J Med Internet Res.

[69] McMullan RD, Berle D, Arnáez S, Starcevic V (2019). The relationships between health anxiety, online health information seeking, and cyberchondria: systematic review and meta-analysis. J Affect Disord.

[70] Du X, Witthöft M, Zhang T, Shi C, Ren Z (2023). Interpretation bias in health anxiety: a systematic review and meta-analysis. Psychol Med.

[71] Westerhof GJ, Miche M, Brothers AF (2014). The influence of subjective aging on health and longevity: a meta-analysis of longitudinal data. Psychol Aging.

[72] Seeman TE, Unger JB, McAvay G, Mendes de Leon CF (1999). Self-efficacy beliefs and perceived declines in functional ability: MacArthur studies of successful aging. J Gerontol B Psychol Sci Soc Sci.

[73] Li JB, Dou K, Liang Y (2021). The relationship between presence of meaning, search for meaning, and subjective well-being: a three-level meta-analysis based on the meaning in life questionnaire. J Happiness Stud.

[74] Chen X, Wang N (2025). How does digital literacy affect the health status of senior citizens? Micro-level evidence from the CFPS data. BMC Health Serv Res.

[75] Jing R, Jin G, Guo Y, Zhang Y, Li L (2023). The association between constant and new Internet use and depressive symptoms among older adults in China: the role of structural social capital. Comput Human Behav.

[76] Chatterjee A, Prinz A, Gerdes M, Martinez S (2021). Digital interventions on healthy lifestyle management: systematic review. J Med Internet Res.

