# Supplementary material for: The Relationship Between Electronic Health Literacy and Health-Related Quality of Life Among Chinese Older Adults: Cross-Sectional Study
Source: J Med Internet Res. 2026 Mar 24;28:e84700. doi: 10.2196/84700 (PMC13012895; doi:10.2196/84700)
Supplement: Multimedia Appendix 1 [file jmir-v28-e84700-s001.docx]

Supplements-Tables

**Table S1**. **Mediated analysis results**

| Variables | | *β* | *SE* | 95% *CI* | |
| --- | --- | --- | --- | --- | --- |
|  |  |  |  | Lower | Upper |
| ATOA | EHL | 0.015**^***^** | 0.001 | 0.013 | 0.016 |
|  | Age | -0.002**^*^** | 0.001 | -0.004 | 0.000 |
|  | Ethnicity | -0.048 | 0.027 | -0.100 | 0.004 |
|  | Region | -0.158**^***^** | 0.012 | -0.182 | -0.134 |
|  | Residence | -0.085**^**^** | 0.025 | -0.133 | -0.036 |
|  | Education | 0.031**^***^** | 0.009 | 0.014 | 0.048 |
|  | Spouse | 0.107**^***^** | 0.026 | 0.056 | 0.158 |
|  | Income | 0.001 | 0.009 | -0.017 | 0.018 |
|  | Living | 0.007 | 0.030 | -0.052 | 0.066 |
|  | Grandcare | 0.119**^***^** | 0.022 | 0.075 | 0.162 |
|  | Smoking | -0.054**^*^** | 0.021 | -0.096 | -0.013 |
|  | Exercise | 0.144**^***^** | 0.016 | 0.111 | 0.176 |
|  | Medical insurance | 0.244**^***^** | 0.057 | 0.132 | 0.356 |
|  | Chronic | -0.111**^***^** | 0.007 | -0.125 | -0.097 |
|  | Outpatient | -0.008**^***^** | 0.002 | -0.011 | -0.004 |
|  | Hospitalization | -0.069**^***^** | 0.012 | -0.092 | -0.046 |
|  | R-squared | 0.144 | | | |
| SE | EHL | 0.019**^***^** | 0.001 | 0.017 | 0.020 |
|  | Age | -0.006**^***^** | 0.001 | -0.007 | -0.004 |
|  | Ethnicity | -0.067**^**^** | 0.020 | -0.107 | -0.028 |
|  | Region | -0.071**^***^** | 0.009 | -0.089 | -0.053 |
|  | Residence | -0.191**^***^** | 0.019 | -0.227 | -0.154 |
|  | Education | 0.047**^***^** | 0.007 | 0.034 | 0.059 |
|  | Spouse | 0.076**^***^** | 0.020 | 0.038 | 0.115 |
|  | Income | 0.041**^***^** | 0.007 | 0.028 | 0.055 |
|  | Living | 0.055**^*^** | 0.023 | 0.010 | 0.100 |
|  | Grandcare | 0.057**^**^** | 0.017 | 0.024 | 0.090 |
|  | Smoking | 0.073**^***^** | 0.016 | 0.041 | 0.104 |
|  | Exercise | 0.064**^***^** | 0.012 | 0.040 | 0.089 |
|  | Medical insurance | 0.086**^*^** | 0.043 | 0.001 | 0.171 |
|  | Chronic | -0.041**^***^** | 0.006 | -0.052 | -0.031 |
|  | Outpatient | -0.003**^*^** | 0.001 | -0.005 | 0.000 |
|  | Hospitalization | -0.022**^*^** | 0.009 | -0.040 | -0.005 |
|  | R-squared | 0.200 | | | |
| PH | EHL | 0.128**^***^** | 0.022 | 0.086 | 0.170 |
|  | ATOA | 3.270**^***^** | 0.265 | 2.752 | 3.789 |
|  | SE | 5.129**^***^** | 0.348 | 4.447 | 5.812 |
|  | Age | 0.048**^*^** | 0.024 | 0.001 | 0.094 |
|  | Ethnicity | -6.256**^***^** | 0.610 | -7.452 | -5.060 |
|  | Region | -6.616**^***^** | 0.283 | -7.171 | -6.061 |
|  | Residence | -5.709**^***^** | 0.567 | -6.821 | -4.597 |
|  | Education | 0.949**^***^** | 0.197 | 0.563 | 1.336 |
|  | Spouse | -0.560 | 0.594 | -1.724 | 0.604 |
|  | Income | 0.462**^*^** | 0.206 | 0.059 | 0.865 |
|  | Living | 0.267 | 0.685 | -1.076 | 1.611 |
|  | Grandcare | 0.725 | 0.510 | -0.275 | 1.725 |
|  | Smoking | -0.446 | 0.484 | -1.394 | 0.502 |
|  | Exercise | 1.240**^**^** | 0.377 | 0.501 | 1.980 |
|  | Medical insurance | -0.132 | 1.304 | -2.688 | 2.424 |
|  | Chronic | -2.314**^***^** | 0.169 | -2.645 | -1.982 |
|  | Outpatient | -0.248**^***^** | 0.039 | -0.324 | -0.172 |
|  | Hospitalization | -3.819**^***^** | 0.269 | -4.347 | -3.291 |
|  | R-squared | 0.237 | | | |
| MH | EHL | -0.069**^**^** | 0.023 | -0.115 | -0.024 |
|  | ATOA | 9.417**^***^** | 0.287 | 8.855 | 9.980 |
|  | SE | 6.929**^***^** | 0.378 | 6.188 | 7.670 |
|  | Age | 0.126**^***^** | 0.026 | 0.076 | 0.177 |
|  | Ethnicity | 0.327 | 0.662 | -0.971 | 1.624 |
|  | Region | -3.618**^***^** | 0.307 | -4.220 | -3.016 |
|  | Residence | -1.016 | 0.615 | -2.222 | 0.191 |
|  | Education | 0.376 | 0.214 | -0.043 | 0.796 |
|  | Spouse | 0.637 | 0.644 | -0.626 | 1.901 |
|  | Income | 1.335**^***^** | 0.223 | 0.897 | 1.772 |
|  | Living | -0.117 | 0.744 | -1.574 | 1.341 |
|  | Grandcare | 0.557 | 0.553 | -0.528 | 1.642 |
|  | Smoking | -2.105**^***^** | 0.525 | -3.133 | -1.076 |
|  | Exercise | 3.096**^***^** | 0.409 | 2.293 | 3.898 |
|  | Medical insurance | 0.843 | 1.415 | -1.930 | 3.616 |
|  | Chronic | -0.728**^***^** | 0.183 | -1.087 | -0.368 |
|  | Outpatient | -0.001 | 0.042 | -0.084 | 0.081 |
|  | Hospitalization | -0.521 | 0.292 | -1.094 | 0.052 |
|  | R-squared | 0.284 | | | |
| LS | EHL | 0.000 | 0.006 | -0.012 | 0.012 |
|  | ATOA | 2.583**^***^** | 0.074 | 2.437 | 2.728 |
|  | SE | 2.250**^***^** | 0.098 | 2.033 | 2.416 |
|  | Age | 0.071**^***^** | 0.007 | 0.058 | 0.084 |
|  | Ethnicity | -0.615**^***^** | 0.171 | -0.950 | -0.279 |
|  | Region | -1.402**^***^** | 0.079 | -1.557 | -1.246 |
|  | Residence | -1.908**^***^** | 0.159 | -2.219 | -1.596 |
|  | Education | 0.099 | 0.055 | -0.010 | 0.207 |
|  | Spouse | 0.416**^*^** | 0.167 | 0.090 | 0.742 |
|  | Income | 0.507**^***^** | 0.058 | 0.394 | 0.620 |
|  | Living | -0.099 | 0.192 | -0.476 | 0.278 |
|  | Grandcare | -0.170 | 0.143 | -0.451 | 0.110 |
|  | Smoking | -0.716**^***^** | 0.136 | -0.982 | -0.450 |
|  | Exercise | 0.539**^***^** | 0.106 | 0.332 | 0.746 |
|  | Medical insurance | 1.236**^**^** | 0.366 | 0.519 | 1.952 |
|  | Chronic | -0.194**^***^** | 0.047 | -0.287 | -0.101 |
|  | Outpatient | 0.004 | 0.011 | -0.018 | 0.025 |
|  | Hospitalization | -0.035 | 0.076 | -0.184 | 0.113 |
|  | R-squared | 0.341 | | | |

**^*^***P*<.05 **^**^***P*<.01 **^***^***P*<.001. PH: physical health; MH: mental health; LS: life satisfaction; EHL: electronic health literacy; ATOA: attitudes toward own aging; SE: self-efficacy.

**Table S2. Age heterogeneity test results**

| Variables | ≤ 74 years | | | > 74 years | | |
| --- | --- | --- | --- | --- | --- | --- |
|  | PH | MH | LS | PH | MH | LS |
| EHL | 0.288**^***^**  (0.026) | 0.188**^***^**  (0.030) | 0.089**^***^**  (0.008) | 0.222**^***^**  (0.036) | 0.175**^***^**  (0.043) | 0.060**^***^**  (0.012) |
| Ethnicity | -7.176**^***^**  (0.746) | -0.247  (0.870) | -0.914**^***^**  (0.225) | -4.948**^***^**  (1.154) | -1.690  (1.380) | -0.636  (0.380) |
| Region |  |  |  |  |  |  |
| Central | -7.315**^***^**  (0.669) | -5.995**^***^**  (0.780) | -2.596**^***^**  (0.202) | -7.203**^***^**  (0.976) | -11.806**^***^**  (1.167) | -2.949**^***^**  (0.322) |
| Western | -16.484**^***^**  (0.775) | -10.859**^***^**  (0.904) | -3.577**^***^**  (0.234) | -12.523**^***^**  (1.144) | -11.966**^***^**  (1.367) | -4.295**^***^**  (0.377) |
| Residence | -6.598**^***^**  (0.749) | -3.754**^***^**  (0.874) | -2.082**^***^**  (0.226) | -6.879**^***^**  (1.243) | -6.914**^***^**  (1.487) | -3.180**^***^**  (0.410) |
| Education |  |  |  |  |  |  |
| Primary school | -0.830  (0.708) | -0.789  (0.826) | -0.460**^*^**  (0.213) | 0.175  (0.882) | -1.532  (1.055) | 0.150  (0.291) |
| Junior school | 0.256  (0.689) | -0.386  (0.804) | -0.276  (0.208) | 4.468**^***^**  (1.050) | 3.114**^*^**  (1.256) | 0.575  (0.346) |
| Senior/Vocational school | 4.912**^***^**  (0.804) | 3.346**^***^**  (0.938) | 0.768**^**^**  (0.242) | 3.286**^**^**  (1.164) | 1.576  (1.392) | 0.621  (0.384) |
| Bachelor and above | 9.452**^***^**  (1.604) | 6.083**^**^**  (1.871) | 1.465**^**^**  (0.484) | 0.478  (1.607) | 2.152  (1.921) | 0.847  (0.530) |
| Spouse | 0.037  (0.857) | 2.610**^**^**  (0.999) | 0.800**^**^**  (0.258) | 0.271  (0.827) | 1.403  (0.989) | 0.730**^**^**  (0.273) |
| Income |  |  |  |  |  |  |
| ≤1000 | -0.171  (0.727) | 1.075  (0.849) | 1.912**^***^**  (0.219) | 2.421**^*^**  (1.015) | 3.787**^**^**  (1.213) | 2.482**^***^**  (0.335) |
| 1001–2000 | 1.682**^*^**  (0.858) | 2.570**^*^**  (1.001) | 1.546**^***^**  (0.259) | 2.230  (1.211) | 1.081  (1.447) | 0.976**^*^**  (0.399) |
| 2001–5000 | 0.398  (0.898) | 6.495**^***^**  (1.048) | 1.846**^***^**  (0.271) | 4.057**^**^**  (1.395) | 7.168**^***^**  (1.668) | 2.601**^***^**  (0.460) |
| ≥5001 | 1.864  (1.118) | 4.681**^***^**  (1.305) | 2.337**^***^**  (0.337) | 4.405**^**^**  (1.572) | 7.384**^***^**  (1.880) | 3.644**^***^**  (0.518) |
| Living | 0.142  (1.003) | 0.331  (1.170) | -0.264  (0.303) | 0.682  (0.953) | -0.024  (1.139) | 0.180  (0.314) |
| Grandcare | 1.184**^*^**  (0.577) | 2.427**^***^**  (0.673) | 0.308  (0.174) | 1.998  (1.340) | 1.631  (1.602) | -0.086  (0.442) |
| Smoking | -0.098  (0.590) | -1.475**^*^**  (0.688) | -0.594**^***^**  (0.178) | -0.848  (0.963) | -2.160  (1.151) | -0.568  (0.317) |
| Exercise | 0.487  (0.483) | 4.098**^***^**  (0.564) | 0.841**^***^**  (0.146) | 5.322**^***^**  (0.640) | 6.617**^***^**  (0.765) | 1.528**^***^**  (0.211) |
| Medical insurance | 0.334  (1.836) | 5.056**^*^**  (2.142) | 1.624**^**^**  (0.554) | 2.487  (1.885) | 3.347  (2.254) | 2.700**^***^**  (0.622) |
| Chronic | -2.583**^***^**  (0.224) | -2.081**^***^**  (0.261) | -0.590**^***^**  (0.068) | -3.331**^***^**  (0.258) | -1.754**^***^**  (0.308) | -0.446**^***^**  (0.085) |
| Outpatient | -0.354**^***^**  (0.052) | -0.106  (0.060) | -0.022  (0.016) | -0.194**^**^**  (0.062) | -0.022  (0.075) | 0.004  (0.021) |
| Hospitalization | -4.756**^***^**  (0.362) | -1.262**^**^**  (0.422) | -0.250**^*^**  (0.109) | -3.237**^***^**  (0.416) | -1.235**^*^**  (0.497) | -0.286**^*^**  (0.137) |
| Observations | 5745 | 5745 | 5745 | 2619 | 2619 | 2619 |
| R-squared | 0.193 | 0.108 | 0.149 | 0.209 | 0.152 | 0.182 |

**^*^***P*<.05 **^**^***P*<.01 **^***^***P*<.001. Estimates are adjusted for other covariates. Robust standard errors are shown in parentheses. Associations between EHL and PH, MH, and LS were significant across different age subgroups identified by the Benjamini-Hochberg procedure (all *P*_(BH-FDR) <.05).

**Table S3. Residence heterogeneity test results**

| Variables | Rural | | | Urban | | |
| --- | --- | --- | --- | --- | --- | --- |
|  | PH | MH | LS | PH | MH | LS |
| EHL | 0.215**^***^**  (0.023) | 0.028  (0.035) | 0.019**^*^**  (0.009) | 0.237**^***^**  (0.032) | 0.391**^***^**  (0.040) | 0.159**^***^**  (0.011) |
| Age | -0.023  (0.030) | 0.114**^**^**  (0.042) | 0.066**^***^**  (0.011) | 0.034  (0.040) | 0.089**^*^**  (0.042) | 0.051**^***^**  (0.011) |
| Ethnicity | 0.883  (0.718) | -0.830  (1.000) | -0.764**^**^**  (0.262) | -15.276**^***^**  (1.058) | -0.626  (1.090) | -0.784**^**^**  (0.286) |
| Region |  |  |  |  |  |  |
| Central | -4.833**^***^**  (0.494) | -7.915**^***^**  (0.689) | -2.783**^***^**  (0.181) | -7.093  (3.655) | -1.374  (3.766) | 1.170  (0.987) |
| Western | 0.335  (1.263) | -8.481**^***^**  (1.760) | -2.017**^***^**  (0.461) | -19.206**^***^**  (0.867) | -11.054**^***^**  (0.894) | -3.945**^***^**  (0.234) |
| Education |  |  |  |  |  |  |
| Primary school | 0.617  (0.516) | -0.098  (0.719) | 0.153  (0.189) | -6.502**^***^**  (1.643) | -3.378**^*^**  (1.693) | -0.856  (0.444) |
| Junior school | 1.171**^*^**  (0.588) | 1.406  (0.819) | 0.338  (0.215) | -2.843  (1.465) | -1.475  (1.509) | -0.410  (0.396) |
| Senior/Vocational school | 2.600**^**^**  (0.869) | 4.858**^***^**  (1.210) | 1.328**^***^**  (0.317) | 0.491  (1.464) | 0.167  (1.508) | 0.105  (0.395) |
| Bachelor and above | 11.367**^***^**  (2.959) | 13.212**^**^**  (4.123) | 4.300**^***^**  (1.081) | -0.688  (1.800) | 0.545  (1.854) | 0.126  (0.486) |
| Spouse | 1.241  (0.660) | 3.289**^***^**  (0.920) | 0.818**^***^**  (0.241) | -1.393  (1.084) | 1.142  (1.117) | 0.992**^***^**  (0.293) |
| Income |  |  |  |  |  |  |
| ≤1000 | 0.921  (0.516) | 1.222  (0.718) | 2.046**^***^**  (0.188) | -0.178  (3.000) | 4.817  (3.091) | 0.460  (0.810) |
| 1001–2000 | 0.494  (0.644) | 1.677  (0.897) | 1.386**^***^**  (0.235) | 6.881**^**^**  (2.239) | 7.738**^***^**  (2.307) | 1.233**^*^**  (0.605) |
| 2001–5000 | 3.607**^***^**  (0.872) | 5.876**^***^**  (1.215) | 2.259**^***^**  (0.318) | 0.050  (1.956) | 11.658**^***^**  (2.015) | 1.632**^**^**  (0.528) |
| ≥5001 | 2.476  (1.339) | 2.607  (1.865) | 2.052**^***^**  (0.489) | 0.231  (2.066) | 10.418**^***^**  (2.129) | 2.205**^***^**  (0.558) |
| Living | 0.460  (0.765) | -0.173  (1.067) | -0.269  (0.280) | 0.278  (1.246) | 1.063  (1.284) | 0.364  (0.337) |
| Grandcare | 0.424  (0.542) | 2.892**^***^**  (0.755) | 0.577**^**^**  (0.198) | 3.346**^***^**  (1.016) | 1.129  (1.047) | -0.137  (0.274) |
| Smoking | -0.468  (0.529) | -2.598**^***^**  (0.738) | -0.856**^***^**  (0.193) | 0.246  (0.944) | 0.082  (0.973) | -0.121  (0.255) |
| Exercise | 1.832**^***^**  (0.416) | 3.525**^***^**  (0.580) | 0.941**^***^**  (0.152) | 5.029**^***^**  (0.720) | 6.504**^***^**  (0.742) | 1.093**^***^**  (0.195) |
| Medical insurance | 2.892**^*^**  (1.242) | 4.072**^*^**  (1.731) | 2.016**^***^**  (0.454) | -3.496  (3.519) | 3.580  (3.626) | 2.634**^**^**  (0.951) |
| Chronic | -3.525**^***^**  (0.212) | -0.929**^**^**  (0.296) | -0.272**^***^**  (0.077) | -2.229**^***^**  (0.267) | -2.681**^***^**  (0.275) | -0.747**^***^**  (0.072) |
| Outpatient | -0.402**^***^**  (0.046) | -0.181**^**^**  (0.065) | -0.045**^**^**  (0.017) | -0.183**^**^**  (0.067) | -0.016  (0.069) | 0.013  (0.018) |
| Hospitalization | -4.619**^***^**  (0.282) | -1.427**^***^**  (0.393) | -0.291**^**^**  (0.103) | -2.829**^***^**  (0.551) | -1.129**^*^**  (0.567) | -0.275  (0.149) |
| Observations | 4726 | 4726 | 4726 | 3638 | 3638 | 3638 |
| R-squared | 0.263 | 0.093 | 0.128 | 0.206 | 0.164 | 0.229 |

**^*^***P*<.05 **^**^***P*<.01 **^***^***P*<.001. Estimates are adjusted for other covariates. Robust standard errors are shown in parentheses. After FDR correction using the Benjamini-Hochberg procedure, the statistical significance conclusions for all subgroup tests remained consistent with the uncorrected results.

**Table S4. Exercise heterogeneity test results**

| Variables | Non-exercisers | | | Exercisers | | |
| --- | --- | --- | --- | --- | --- | --- |
|  | PH | MH | LS | PH | MH | LS |
| EHL | 0.322**^***^**  (0.033) | 0.196**^***^**  (0.047) | 0.067**^***^**  (0.012) | 0.193**^***^**  (0.023) | 0.147**^***^**  (0.032) | 0.077**^***^**  (0.008) |
| Age | -0.023  (0.040) | 0.112**^*^**  (0.050) | 0.066**^***^**  (0.013) | 0.046  (0.031) | 0.075**^*^**  (0.035) | 0.047**^***^**  (0.009) |
| Ethnicity | -4.777**^***^**  (0.940) | 2.199  (1.176) | -0.246  (0.303) | -7.966**^***^**  (0.849) | -3.280**^***^**  (0.955) | -1.218**^***^**  (0.256) |
| Region |  |  |  |  |  |  |
| Central | -6.459**^***^**  (0.835) | -7.342**^***^**  (1.044) | -2.976**^***^**  (0.269) | -7.813**^***^**  (0.746) | -7.942**^***^**  (0.839) | -2.329**^***^**  (0.224) |
| Western | -9.378**^***^**  (1.183) | -6.415**^***^**  (1.480) | -1.685**^***^**  (0.381) | -18.536**^***^**  (0.766) | -13.597**^***^**  (0.861) | -4.777**^***^**  (0.230) |
| Residence | -10.623**^***^**  (1.035) | -8.876**^***^**  (1.294) | -3.810**^***^**  (0.333) | -2.165**^**^**  (0.833) | -0.419  (0.937) | -1.025**^***^**  (0.251) |
| Education |  |  |  |  |  |  |
| Primary school | 0.969  (0.888) | -0.219  (1.111) | 0.134  (0.286) | -1.096  (0.708) | -1.517  (0.796) | -0.463**^*^**  (0.213) |
| Junior school | 2.818**^**^**  (0.956) | 3.558**^**^**  (1.196) | 0.605**^*^**  (0.308) | 0.615  (0.718) | -1.284  (0.807) | -0.310  (0.216) |
| Senior/Vocational school | 2.946**^*^**  (1.153) | 6.037**^***^**  (1.442) | 1.702**^***^**  (0.371) | 4.416**^***^**  (0.810) | 0.994  (0.911) | 0.321  (0.244) |
| Bachelor and above | 2.126  (2.077) | 10.678**^***^**  (2.598) | 2.637**^***^**  (0.669) | 4.592**^***^**  (1.320) | 0.434  (1.485) | 0.361  (0.397) |
| Spouse | 2.719**^**^**  (0.961) | 3.175**^**^**  (1.202) | 0.994**^**^**  (0.310) | -1.671**^*^**  (0.779) | 1.550  (0.876) | 0.786**^***^**  (0.234) |
| Income |  |  |  |  |  |  |
| ≤1000 | 2.168**^*^**  (0.908) | 2.956**^**^**  (1.136) | 2.430**^***^**  (0.293) | -0.805  (0.777) | 0.959  (0.875) | 1.810**^***^**  (0.234) |
| 1001–2000 | 2.920**^*^**  (1.219) | 1.458  (1.525) | 1.253**^**^**  (0.393) | 0.493  (0.862) | 2.049**^*^**  (0.969) | 1.303**^***^**  (0.259) |
| 2001–5000 | 3.210**^**^**  (1.222) | 6.380**^***^**  (1.529) | 2.422**^***^**  (0.394) | -1.430  (0.960) | 5.151**^***^**  (1.079) | 1.270**^***^**  (0.289) |
| ≥5001 | 5.718**^***^**  (1.502) | 3.770**^*^**  (1.879) | 2.873**^***^**  (0.484) | -1.297  (1.129) | 4.584**^***^**  (1.270) | 1.968**^***^**  (0.340) |
| Living | 1.966  (1.164) | 0.342  (1.456) | -0.079  (0.375) | -1.027  (0.876) | 0.155  (0.986) | 0.054  (0.264) |
| Grandcare | 0.381  (0.926) | 2.450**^*^**  (1.158) | 0.780**^**^**  (0.298) | 1.880**^**^**  (0.624) | 2.028**^**^**  (0.701) | -0.019  (0.188) |
| Smoking | 0.165  (0.794) | -1.788  (0.993) | -0.454  (0.256) | -0.307  (0.641) | -1.209  (0.721) | -0.608**^**^**  (0.193) |
| Medical insurance | 2.747  (2.666) | 4.510  (3.334) | 2.088**^*^**  (0.859) | 0.616  (1.514) | 4.055**^*^**  (1.703) | 2.023**^***^**  (0.455) |
| Chronic | -4.215**^***^**  (0.298) | -3.156**^***^**  (0.373) | -0.776**^***^**  (0.096) | -2.093**^***^**  (0.207) | -1.263**^***^**  (0.233) | -0.422**^***^**  (0.062) |
| Outpatient | -0.323**^***^**  (0.072) | -0.028  (0.090) | 0.018  (0.023) | -0.322**^***^**  (0.048) | -0.148**^**^**  (0.054) | -0.036**^*^**  (0.014) |
| Hospitalization | -3.949**^***^**  (0.429) | -1.328**^*^**  (0.537) | -0.460**^***^**  (0.138) | -4.144**^***^**  (0.358) | -1.140**^**^**  (0.402) | -0.096  (0.108) |
| Observations | 3127 | 3127 | 3127 | 5237 | 5237 | 5237 |
| R-squared | 0.228 | 0.110 | 0.158 | 0.189 | 0.098 | 0.154 |

**^*^***P*<.05 **^**^***P*<.01 **^***^***P*<.001. Estimates are adjusted for other covariates. Robust standard errors are shown in parentheses. The association between EHL and PH, MH, and LS was significant across different exercise habit subgroups identified by the Benjamini-Hochberg procedure (all *P*_(BH-FDR) <.05).
